# The Tenth Semi-Annual Meeting of the South Texas Medical Association, Houston, Texas, Thursday and Friday, June 20 and 21, 1901

**Published:** 1901-06

**Authors:** 


### The Tenth Semi-Annual Meeting of the South Texas Medical Association, Houston, Texas, Thursday and Friday, June 20 and 21, 1901.

### ORDER OF EXERCISES.

Call to order at 10:30 o'clock by Dr. R. W. Knox, President South Texas Medical Association.

Invocation by Rev. H. A. Aves, Rector Christ Church.

Address of welcome by the President.

### SECTION WORK.—FIRST DAY.

#### SECTION ON SURGERY.

Dr. J. E. Thompson, Galveston, Chairman.

1. "Scraps of Surgical Cases," Dr. Frank B. King, Houston. 2. "A Case of Aneurysmal Varix," Dr. J. W. Thomason, Huntsville. 3. "Report of a Case of Phelps' Open Operation for Equinovarus," Dr. Walter Shropshire, Yoakum. 4. "The Surgical and Medical Anatomy of the Stomach with Three Cases of Cancer of that Viscus," Dr. Wm. Keiller, Galveston. 5. "Abdominal Surgery in Private Homes," Dr. J. B. York, Brenham. 6. "Analysis of Nine Cases of Nephrectomy," Dr. James E. Thompson, Galveston. 7. "Cranial Fractures," Dr. S. C. Red, Houston. 8. "Intra-peritoneal Retro-uterine Hematoma—Report of a Case," Dr. H. A. Barr, Beaumont.

SECTION ON PRACTICE.

Dr. Walter T. Brown, Wallisville, Chairman.

1. "Infant Feeding," Dr. James H. Bute, Houston. 2. "Ovarian and Uterine Reflexes," Dr. J. H. Reuss, Cuero. 3. "Treatment of Acute Dysentery with Sulphate of Magnesia," Dr. Ira T. Clemons, Monaville. 4. "My Experience and Treatment in Eight Cases of Typhoid Fever," Dr. C. A. H. Arnecke, Arneckeville. 5. "Two Cases of Hemaglobinuric Fever—Report of Two Cases," Dr. Walter Shrophire, Yoakum. 6. Subject Unannounced, Dr. E. A. Malch, Edna. 7. "Specific Urethritis," Dr. G. D. Parker, Houston. 8. "Palatable Prescribing for Children," Dr. R. W. Knox, Houston. 9. "Medical Reorganization in the United States with Special Reference to Texas." 9. "Infant Feeding," Dr. E. J. Hamilton, Houston.

SECTION ON OPHTHALMOLOGY.

Dr. E. P. Daviss, Chairman.

1. "Report of a Case of Cataract in a Patient age 98, and Exhibition of a New Snare for Tonsil Operations," Dr. G. P. Hall, Galveston. 2. "Strabismus and Its Treatment," Dr. F. W. Kirkham, Cuero. 3. "Prophylaxis of the Special Senses in the Exanthemata of Childhood," Dr. J. A. Mullen, Houston. 4. "Exophoria; Its Significance and Treatment," Dr. Henry C. Haden, Philadelphia.

SECTION ON OBSTETRICS AND GYNECOLOGY.

Dr. R. T. Morris, Chairman.

1. "Report of Two Cases," Dr. J. W. Scott, Houston. 2. "The Surgical Technique of Minor Gynecology," Dr. R. T. Morris, Houston. 3. Subject Unannounced, Dr. Donald McKay, Houston. 4. "Some Remarks on Perineorrhaphy with Special Reference to the Technique of Deep Sutures," Dr. O. L. Norseworthy, Houston.

